# Association of lung-intestinal microecology and lung cancer therapy

**DOI:** 10.1186/s13020-023-00742-8

**Published:** 2023-04-10

**Authors:** Ling-Yu Kong, Xuan-Yu Chen, Xin Lu, Qinggele Caiyin, Dong-Hua Yang

**Affiliations:** 1 School of Chemical Engineering and Technology, Tianjin University, Tianjin, China; 2 Traditional Chinese and Western Medicine Oncology Clinic, North China University of Science and Technology Affiliated Hospital, Tangshan, Hebei China; 3 Institute for Biotechnology, St. John’s University, Queens, NY 11439 USA; 4 Clinical School of Medicine, North China University of Science and Technology, Tangshan, Hebei China; 5 New York College of Traditional Chinese Medicine, Mineola, NY 11501 USA

**Keywords:** Lung cancer, Lung-gut microecology, Cancer metastasis, Gut microbiota, Lung-gut co-treatment

## Abstract

In recent years, the incidence of lung cancer is increasing. Lung cancer has become one of the most malignant tumors with the highest incidence in the world, which seriously affects people’s health. The most important cause of death of lung cancer is metastasis. Therefore, it is crucial to understand the mechanism of lung cancer progression and metastasis. This review article discusses the physiological functions, pathological states and disorders of the lung and intestine based on the concepts of traditional Chinese medicine (TCM), and analyzes the etiology and mechanisms of lung cancer formation from the perspective of TCM. From the theory of “the exterior and interior of the lung and gastrointestinal tract”, the theory of “the lung-intestinal axis” and the progression and metastasis of lung cancer, we proposed e “lung-gut co-treatment” therapy for lung cancer. This study provides ideas for studying the mechanism of lung cancer and the comprehensive alternative treatment for lung cancer patients.

## Introduction

Over the past decades, lung cancer has persisted as one of the most common cancers in both men and women worldwide [1, 2]. Although progress in its diagnosis and treatment has been made in recent years, lung cancer still accounts for a high proportion of cancer-related deaths, and the 5-year survival rate is low [3, 4]. Recent studies suggest that intestinal flora have immune functions in addition to its barrier role in the gastrointestinal tract, and that dysbiosis of the intestinal flora is associated with inflammatory and malignant diseases of the gastrointestinal system. In recent years, it has been shown that imbalance or dysregulation of the gut microbiota is associated with development of various tumors [5–7]. There is a close relationship between the theory of "the lung and large intestine are external and internal" and the concept of the “lung-gut axis” in clinical medicine. However, the research on lung microecology, intestinal flora, and lung cancer development and treatment are limited. This review article will discuss the connection between "the lung and large intestine are external and internal" and lung-gut microecology, and finally explore the pathogenesis and the “lung-gut co-treatment” therapy to provide evidence for comprehensive alternative therapy for lung cancer.

## The physiological functions of the lung and intestine in traditional Chinese medicine (TCM)

**Fig. 1:**
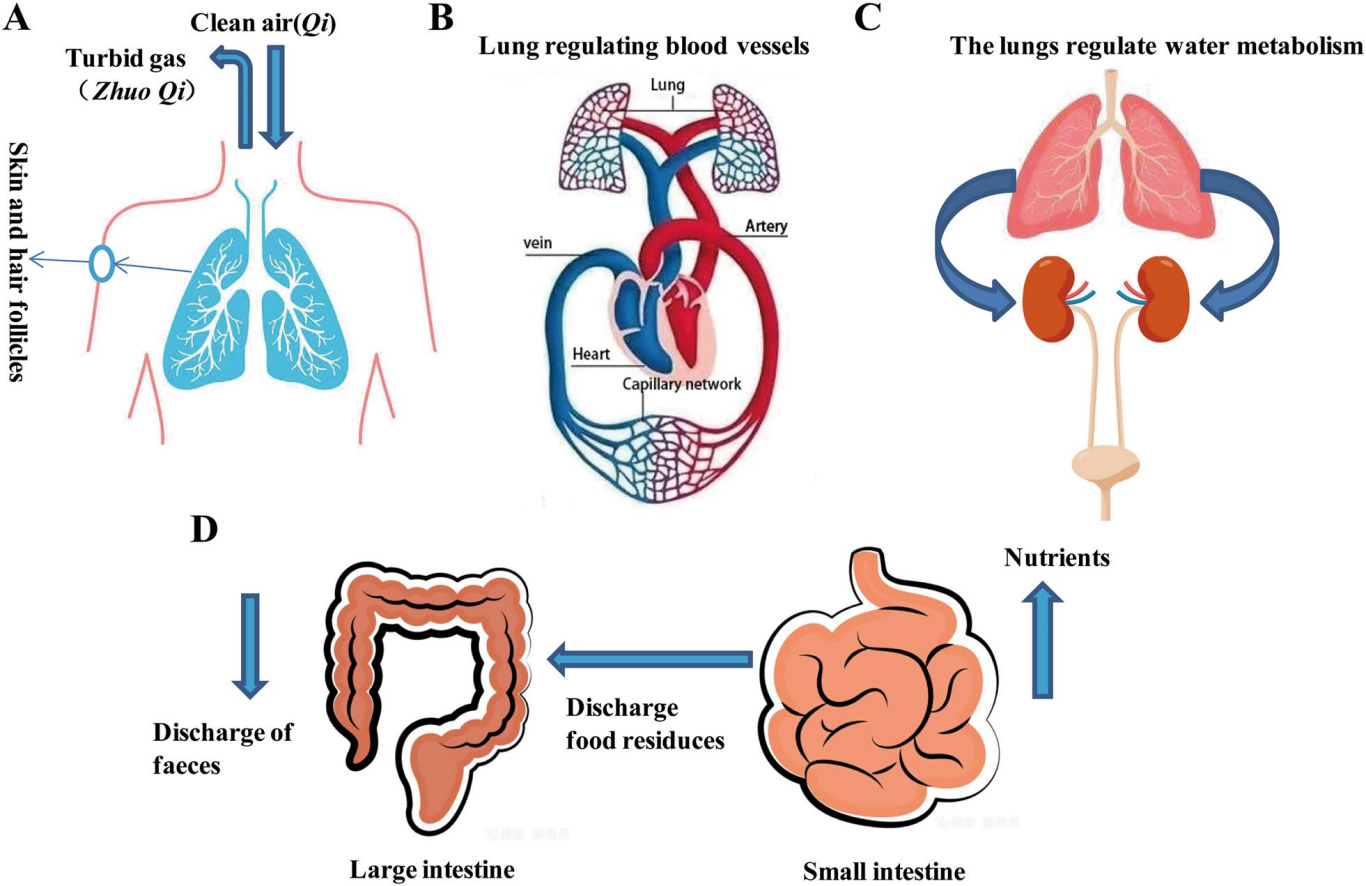
Physiological functions of lung and intestine in Traditional Chinese Medicine. The lungs are responsible for gas exchange and respiration, and are closely related to skin (**A**). Lung governance regulates blood circulation (**B**), The lungs participate the body’s water and fluid metabolism (**C**). Physiological functions of large intestine and small intestine in Traditional Chinese Medicine(**D**).

The physiological functions of the lung and intestine have been documented in ancient Chinese literatures. The main physiological functions of the lung according to TCM (Fig. 1) are: (1) The lung governs *Qi* and is responsible for respiration. This function of the lung includes the propagation of Wei *Qi* (defense *Qi* ). The body needs to continuously inhale clean air from the nature and eliminate turbid gas from the body in the process of metabolism. The lungs are the place of gas exchange in the body. Through the respiratory function of the lungs, the body continuously breathes in and out to maintain its vital activities. The function of the lungs in respiration is dependent on its function in governing gas diffusion and purification. In addition, the main function of the lung is to promote and disperse the *Wei Qi*. The lung is closely related to skin, sweat glands and hair follicle tissue functions, achieving the regulation of the lung Qi to prevent the invasion of external evils from the skin and hair. (2) The lung connects and regulates all blood vessels, which means that the whole body’s blood converges on the lung through hundreds of veins and arteries, and the heart. Through the respiration of the lung, the exchange of clean air in the lungs is carried out, and then the oxygenated blood is carried through the circulation (heart) to the whole body. The ancient Chinese medical book “*Inner Canon of Yellow Emperor*” says that the function of the lungs is equivalent to that of a court chancellor, assisting the monarch (heart) and governing the regulation of of *Qi* in the internal organs and blood. Chinese medicine believes that *Qi*, blood, and fluids are the fundamental substances of the human body, and the lung plays the role of governing and regulating the whole body through governing and regulating *Qi*, blood, and fluids, among them, governing and regulating *Qi* is most critical. (3) The lungs are responsible for regulating the water channels. It means that the lung governs the distribution, operation, and excretion of water and fluid in the body. Through the descent action of the lungs, the water is distributed upward and outward, spreading throughout the body, reaching the skin and hair, and is metabolized and excreted in the form of sweat; the water goes downward and becomes the source of urine production, and through the transpiration and gasification of the kidneys, the metabolized water is liquefied into urine and stored in the bladder, and then eliminated from the body. According to the *Inner Canon of Yellow Emperor*, the large intestine is the organ in charge of transmission and removing solid waste. Chinese medicine believes that the main physiological function of the large intestine is to transmit and transform the dregs. The large intestine accepts the food residues and fluid after digestion in the small intestine. Then it absorbs the remaining fluid to form feces, which is transmitted to the end of the large intestine and eliminated from the body through the anus. The primary physiological function of the small intestine is to digest and absorb foods and to transmit its essence. The small intestine is a vital organ that transmits food residues and plays an essential role in converting food into nutrients [8–10].

## Pathological states and disorders of the lung and intestine

**Fig. 2:**
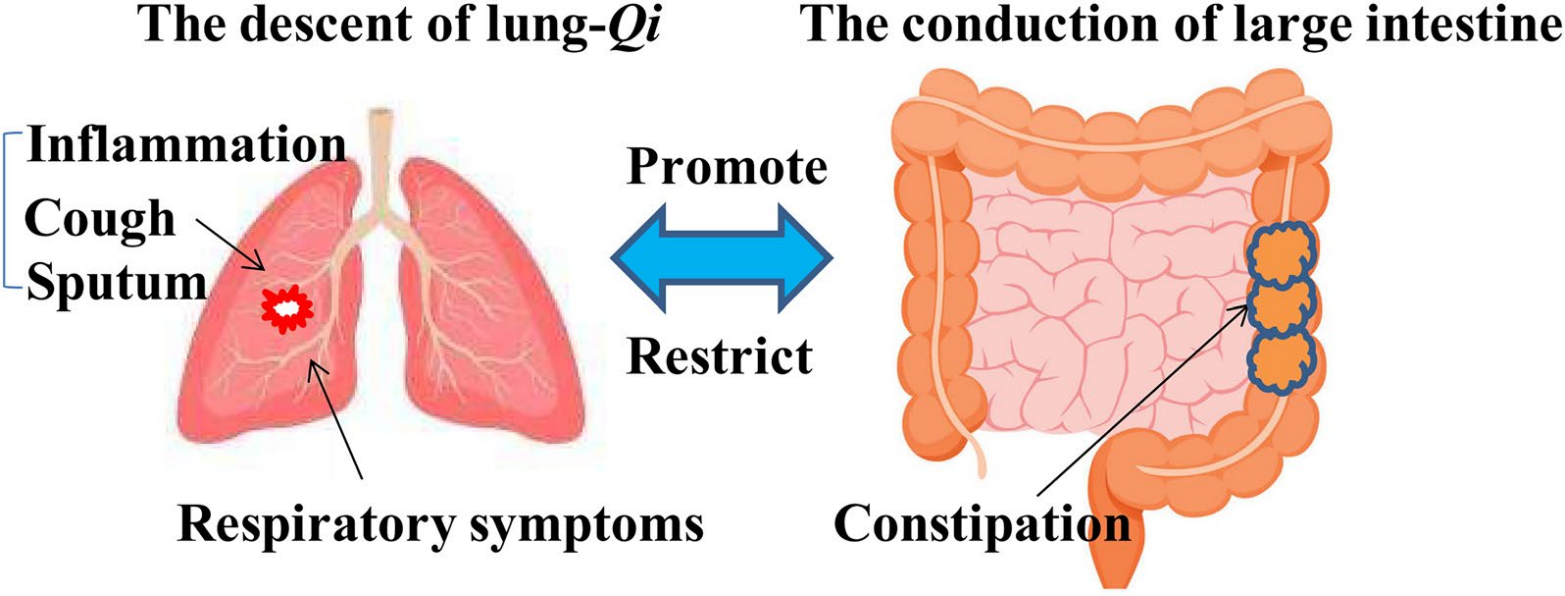
Lung and large intestine are in interior-exterior relationship. Lung and large intestine interacts with each other, lung symptoms can affect the function of the large intestine, and conversely, disorders of the large intestine can affect lung function

*The Inner Canon of Yellow Emperor* said that the lung and the gastrointestinal tract have the same *Qi* in the exterior and interior, so the condition of the large intestine can be reflected in the skin. Lung meridian begins at the heart and lungs and travels down to the large intestine. *Yang* is *Zang* (solid organs, such as the lungs), and the *Yang* meridians travel on the outer side of the hand; *Yin* is *Fu* (hollow organs, such as the gastrointestinal tract), and the *Yin* meridians travel on the inner side of the hand. This is also called the “exterior and interior relationship” of the hand. The lung and large intestine are related to each other through the meridians [11]. The lung governs *Qi*, moves water and disperses fluids; the large intestine governs fluids and enables normal fecal excretion. If the lung *Qi* cannot go down, dry stool and constipation can occur. If the lung *Qi* is weak and unable to push the force, the stool is hard and astringent, so called “*Qi* deficiency constipation”. If the *Qi* and blood cannot be consolidated and the clearing and turbidity are mixed and move downward, diarrhea will occur. The descending of lung *Qi* helps the function of large intestine conduction, and the normal function of large intestine conduction helps the descending function of the lung. Conversely, intestinal disease can transmit to the lung, and if the “evil” (pathogenic factors in TCM) is in the large intestine, or if the large intestine has excess heat and that rushes up to the lung, the lung will be impaired, the internal *Qi* will be blocked, which will affect the diffusion of lung and produce symptoms such as chest fullness and cough [12, 13]. It can be seen that lung and large intestine interact with each other. The lung disease can affect the intestine, and intestine disease affects the lung, and the lung and intestine can become dis-functioning at the same time (Fig. 2).

## The etiology and mechanisms of lung cancer formation in TCM

**Fig. 3:**
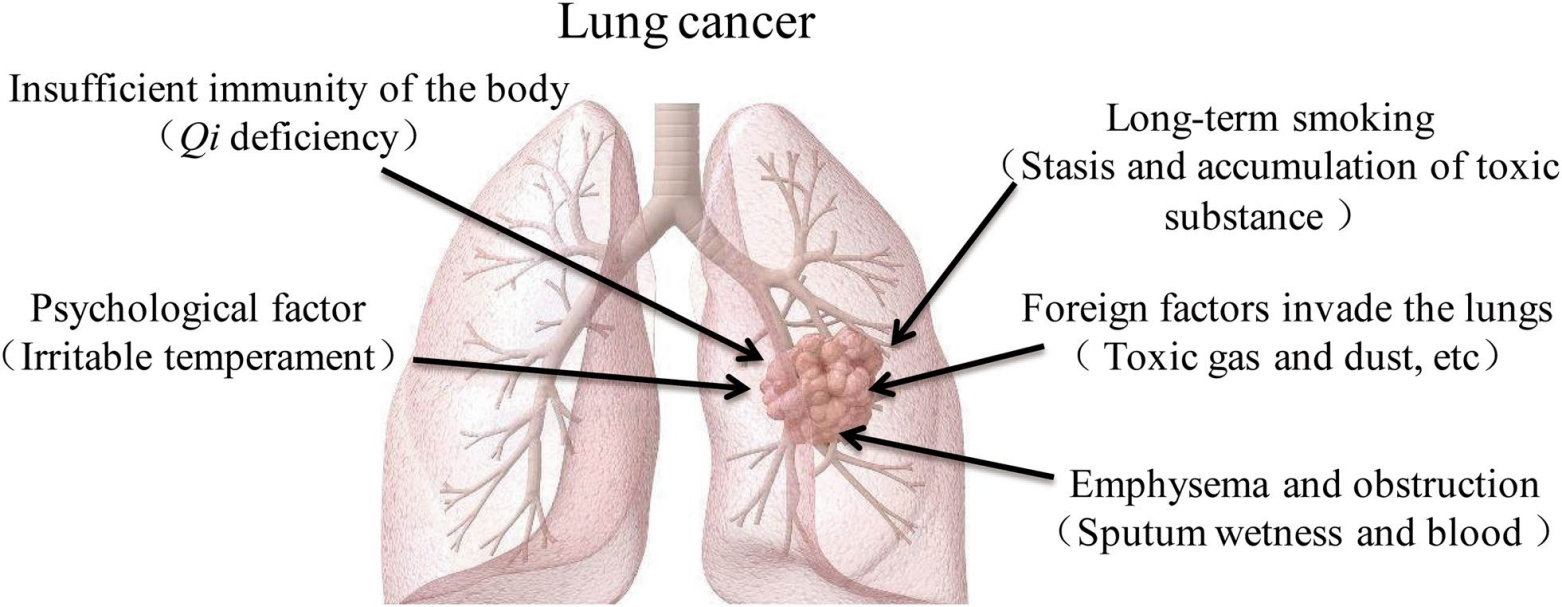
According to the theory of Traditional Chinese Medicine, the cause of the occurrence and formation of lung cancer includes internal factors as well as external ones

The Chinese medical name for lung cancer is "Xiben" *The Inner Canon of Yellow Emperor* states that “the buildup of disease is gradually accumulated and cannot be cured in 2 or 3 years”. *The Genuine Meaning of the Difficult Classic* said that if Xiben is not cured, it will make people sensitive to cold and heat, with symptoms of shortness of breath and cough, and in serious cases, abscess of the lung may occur. Integrating ancient literature and modern concepts, Chinese medicine believes that the formation of lung cancer is mainly due to the following aspects (Fig. 3): First, internal deficiency of *Yang Qi* and imbalance of *Yin* and *Yang* in the internal organs are the main causes of lung cancer. Chronic lung diseases, depletion of lung *Qi*, or overexertion and deficiency of lung *Qi* and lung *Yin* will lead to external evil taking advantage of the deficiency and staying in the lungs, resulting in stagnation of blood flow in the lungs and becoming a mass. The second is injury caused by the seven emotions. Impatience will lead to counterflow of *Qi* and then cause *Qi* stagnation in the lung. The third is the internal accumulation of toxic smoke. Long-term smoking and fluid depletion would result in lung *Yin* deficiency. *Qi* with *Yin* deficiency, coupled with the smoke containing nicotine and tar and other toxic substances, can detain the lung orifices, block the airway, resulting in phlegm and dampness, blood clotting, and the formation of tumors. Fourth, the lung is attacked by evil toxic substances. The lung is a delicate viscus and is susceptible to be attacked by evil toxic substances, such as air pollution, asbestos, ore dust, airborne dust and radioactive substances, which lead to dysregulation of the purification of the lung *Qi*, stagnation of the lung *Qi* and promote blood stasis, resulting in the formation of tumors. Fifth, phlegm and dampness gather in the lungs; the spleen is responsible for transporting water and grain; spleen deficiency and dysregulation of transporting and transforming, and water cannot be transported and distributed, resulting in the production of phlegm from dampness and retention in the lungs, or uncontrolled diet and long-term use of fatty foods, resulting in water and dampness and phlegm, and phlegm gathering in the lungs, leading to stagnation of *Qi* and blood and stagnation in the lungs, and gradual formation of masses [14–17].

## Lung and gastrointestinal microecology in the pathogenesis and treatment of lung cancer

### Respiratory tract microbiota and lung cancer formation

**Fig. 4:**
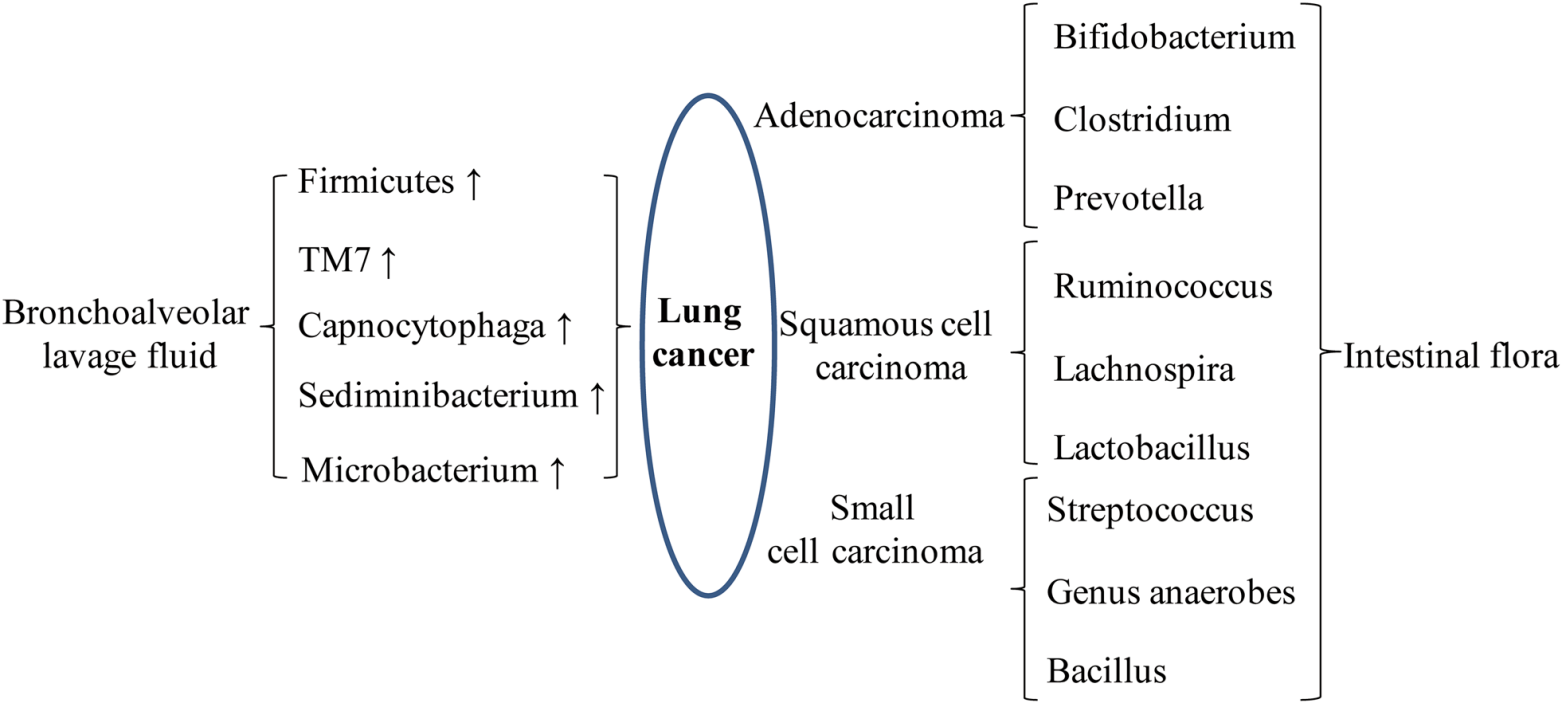
Composition of respiratory tract flora in lung cancer patients and characteristics of intestinal flora in different types of lung cancers. The left side shows the main composition of respiratory microbiota in lung cancer patients, and the right side shows the composition of intestinal microbiota of three common types of lung cancers

New gene sequencing technologies have fundamentally revolutionized the understanding of lung as a sterile organ, suggesting that healthy human lungs are also colonized by bacterial flora [17], which is important for the formation of immune system [18] and the development of tolerance to allergens[19]. The biomass of the pulmonary microbiota is significantly lower than that of the intestinal flora, with approximately 10–100 bacteria per 1000 human cells [20, 21]. The pulmonary flora is a dynamic ecosystem, with the main bacterial phyla in the lungs and intestines of healthy subjects broadly consistent, containing the *Firmicutes (Staphylococcus, Streptococcus, and Lactobacillus)* and the *bacteroidetes*, followed by the *Pseudomonadota* and *Actinomycetes*, which tend to be increased in lung tumors [22–26]. There is a cross-border influence in pulmonary flora formation that may involve several pathways such as physical interactions, population sensing molecules, inflammatory responses, antimicrobial drugs, immune response modulation and nutrient exchange. For example [27], there is a synergistic effect between *Pseudomonas* and *Streptococcus*, where *Pseudomonas* stimulates *Streptococcus* growth, increases biofilm formation, or enhances *Candida* pathogenicity via *Streptococcus*. However, the regulation of the pulmonary flora is not limited to internal associations but also depends on the interaction between the intestine and the lungs. The human mouth and lung are the gateway for many microbial particles and bacteria to enter the human body [25]. There is evidence of an association between the pulmonary microbiota and lung health, and the pulmonary flora is thought to underlie the pathophysiology of many respiratory diseases [28]. The pulmonary flora is altered in many respiratory diseases, such as obstructive lung disease, interstitial lung disease, infectious diseases, and lung cancer. One study found a significant association between *Mycobacterium tuberculosis* (TB) and lung cancer [29, 30], and a possible reason for this is that persistent TB infection can cause production of tumor necrotic factors and lead to lung inflammation, pulmonary fibrosis leading to increase extracellular stroma, and finally lung cancer formation [31, 32]. Several other studies have shown that a low-density, diverse microbial ecosystem is present in bronchoalveolar lavage fluid, sputum, and lung tissue [33]. For example, Lee et al. reported that analysis of bronchoalveolar lavage fluid from 20 lung cancer patients showed that the abundance of two bacteria (Firmicutes and TM7) and two genera were significantly higher in lung cancer patients than in the healthy population [34, 35]. Moreover, TM7-3, *Capnocytophaga*, *Sediminibacterium*, and *Microbacterium* were also significantly enriched compared to controls [36–38] (Fig. 4). Lung cancers of different pathological tissue types are associated with specific microflora in the lung. A study in which sputum and saliva samples from 30 lung cancer patients [39] were analyzed by high-throughput sequencing of the 16 S ribosomal RNA (rRNA), which showed that the genera *Capnocytophaga, Selenomonas, Veillonella*, and *Neisseria* were associated with adenocarcinoma (AC) and small cell carcinoma (SCC), and these genera could be used as biomarkers for the diagnosis of different types of lung cancer. Currently, samples are obtained mainly by means of bronchoalveolar lavage fluid or tissue samples, which does not exclude the possibility of cross-contamination. In addition, studies have shown that the microbiota of the upper and lower airways are different; therefore, the results of this study need validation using larger sample experiments, better sampling methods, and considering the influence of different airway sites [40–42]. Studies on the connection between lung flora and lung cancer are gaining attention, however, there is a lack of exploration of the mechanisms of the role of lung flora in lung carcinogenesis. Large cohort, macrogenomic and metabolomic studies on the mechanisms are needed in the future.

### The association between gastrointestinal flora imbalance and lung cancer

H. pylori infection may be related to the progression and treatment of lung cancer [43]. A retrospective study was conducted in two independent cohorts of PD-1-treated patients with non-small-cell lung cancer (NSCLC). Using enzyme-linked immunosorbent assay (ELISA) to measure serum H. pylori antigen-directed IgG antibodies, H. pylori seropositivity was associated with lower efficacy of anti-PD-1 immunotherapy [44]. The intestinal flora, the “connecting network” of biological metabolism, is a collection of bacteria colonizing the gastrointestinal tract [45, 46]. The intestinal microbiota is a critical component of the host immune response, performing many essential functions for the maintenance of human health. The gastrointestinal tract is the primary site of microbial growth in the human body. A 20–30-year-old person weighing 70 kg is estimated to have approximately 3.8 × 10^13^ bacteria colonizing the gastrointestinal tract, with over 1000 species representing 0.3% of the body weight. The flora composition is similar in most healthy populations, with more than 90% of all bacteria being of the firmicutes and bacteroidetes, followed by the Actinomycetes, Pseudomonadota and Verrucomicrobia, which together constitute 99% of the commensal flora [45, 46]. About 60 genera have been identified as “core” flora, mainly derived from *Bacteroides, Bifidobacterium, Eubacterium, Rumenococcus, and E. faecalis* [47]. Growing evidence suggests a close association of microbiota between the gastrointestinal and respiratory tracts, and that exacerbation of chronic intestinal and pulmonary diseases is associated with disorders and imbalances of microbial ecosystem [48, 49]. Intestinal dysbacteriosis is linked to different lung diseases, such as asthma [50], chronic obstructive pulmonary disease (COPD) [51], and lung cancer [52]. The intestinal flora provides energy by breaking down food and produces major metabolites such as short-chain fatty acids (SCFAs) [53–55]. The concentration of intestinal circulating SCFAs affects the level of IL-6 and IL-8 in lung tissues and is associated with the development of lung cancer [56, 57]. In addition, previous studies have found that butyrate-producing bacteria are dysregulated in the intestine of patients with non-small cell lung cancer [58]. Gut microbiota alpha diversity (number and abundance) and taxa homogeneity were significantly higher in non-malignant lung tissues than in tumor-bearing lung tissues, with no significant differences in beta diversity. *Streptococcus, Prevotella, Blautia, Coprococcus, Bifidobacterium and Helicobocton Pyloni* were found to be enriched in the intestinal flora of lung cancer patients [52, 59]. By analysis of 216 bronchoscopic samples, it was found that Gram-negative bacteria such as *Haemophilus influenzae*, *Enterobacteriaceae* and *Escherichia coli* were found to be prone to colonize in lung cancer tissues [60]. Tsay et al. demonstrated that patients with stage IIb-IV non-small cell lung cancer of the lower airway had more abundant disorders of the intestinal flora, which were associated with upregulation of IL17, PI3K, MAPK and ERK pathways in the airway transcriptome. It was suggested that *Peillonella parvula* plays a major driving role and promotes cancer progression [61]. In addition, microbiota-induced helper T-cell 17 (Th17) can promote lung cancer cell proliferation and angiogenesis[62]. Symbiotic bacteria are essential for γδT17 cell responses in the mouse lung as a potential mechanism for the shortened mean survival time in the Lewis lung cancer model. It has been shown [63, 64] that in the intestinal flora of lung adenocarcinoma patients, *Bifidobacterium*, *Clostridium*, and *Prevotella* were the dominant strains; in lung squamous cell carcinoma patients, a higher proportion of *Bifidobacterium tumefaciens*, *Trichosporon spp.* and *Lactobacillus spp.* were found; while *Streptococcus spp*., *Anaerobes spp.* and *Bacillus spp.* were more frequently shown in small cell lung cancer. These observations suggest that microbiota in the distal gut can also be used as a biomarker for lung cancer (Fig. 4).

## Mechanism of lung-gut axis dysregulation in lung cancer progression

**Fig. 5:**
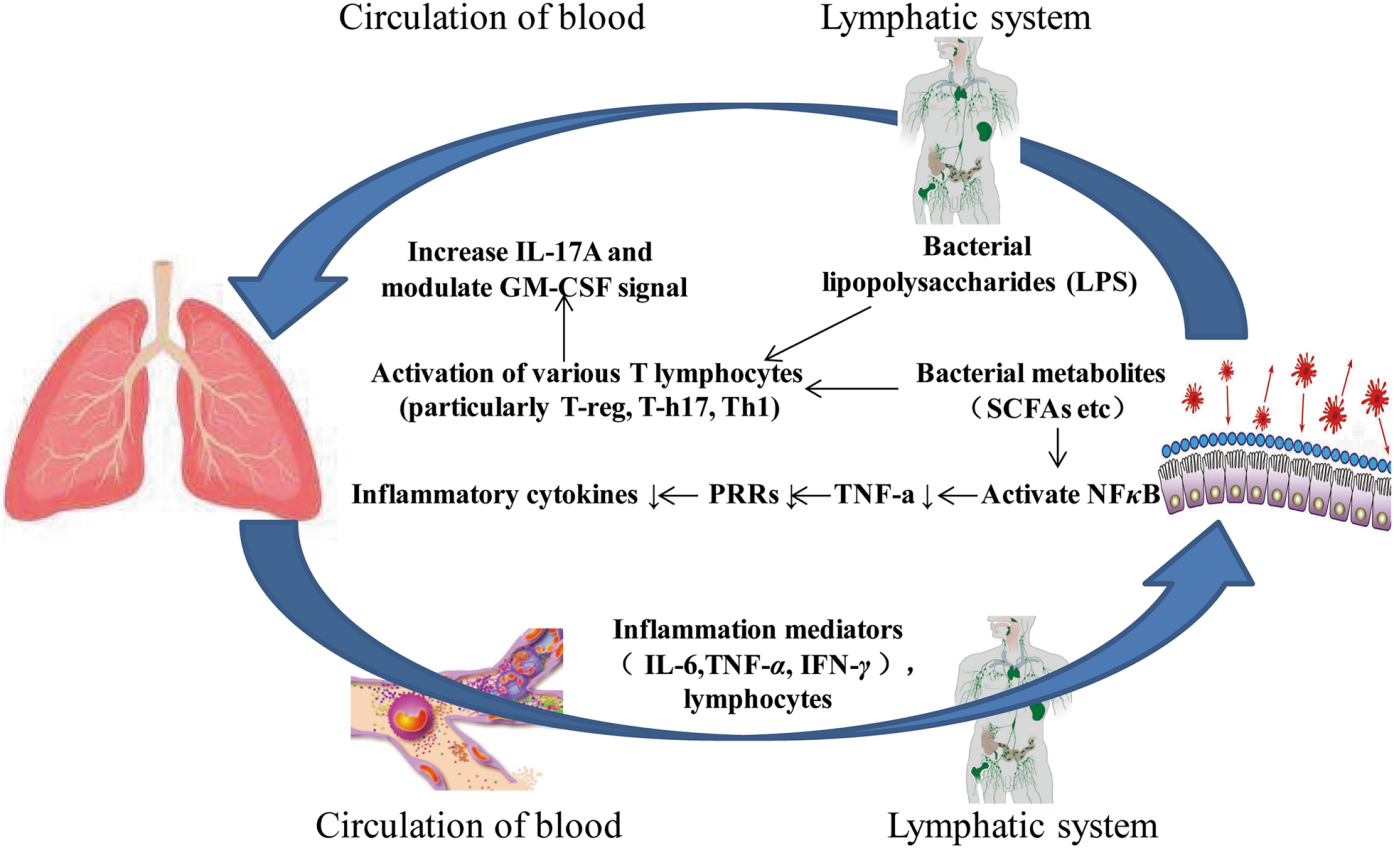
Lung-gut axis theory and mechanisms of lung-gut interaction. The upper part shows the influence of intestinal microecology on lung diseases and the main pathways. The lower part shows pulmonary inflammation affects intestinal function. The lung and gut interact with each other through blood and lymphatic pathways

Although the composition of flora in the intestinal and respiratory tracts differs, the epithelia of both gastrointestinal and respiratory tracts develop structurally from the same embryonic structure, with similar mucosa in both anatomical structure and function. The early microbial colonization of the intestine and lung is similar. Thus, there is growing evidence highlighting the relationship and interactions between the intestine and lung, called the lung-gut axis [65, 66]. The lung-gut axis refers to the interface of immune information between two microbiota systems in the lung and the intestine. The “lung-gut axis” is mediated by inflammation and includes the gut-induced entry of immune cells, cytokines, bacteria, bacterial products, and some other microorganisms across the gastrointestinal barrier and into the body circulation through the blood and lymphatic systems [67]. The gut microbiota can modulate and alter the immune activity of the lung through bacterial lipopolysaccharides (LPS) and various bacterial metabolites (e.g., SCFA and others), which subsequently cause activation of multiple T lymphocytes (especially T-reg, T-h17, and Th1) after stimulation of dendritic cell production and migration to the lower respiratory tract through the circulating bloodstream. In contrast, bacterial metabolites lead to a decrease in tumor necrosis factor (TNF-a) through the mobilization of activated B cells and nuclear factor kappa-light-chain-enhancer (NFκB), which leads to a down-regulation of pattern recognition receptors (PRRs) and thus a decrease in cytokines (interleukin-1, interleukin-12, interleukin-18, TNF-α, IFN-γ and GM-CSF) (Fig. 5). This vital link maintains homeostasis of the pulmonary immune system and vice versa, thus avoiding ecological dysregulation of microorganisms [28, 68–73] .

For that matter, how does the gut influence the progression of lung disease and even lung cancer? Gastrointestinal commensal flora maintains a stable state by regulating the immune function of the digestive system and distant organs, and regulates the T cell population outside the gastrointestinal tract and acts on regulatory T cells (Treg) to produce SCFAs and trigger systemic inflammatory responses. [68]. Intestinal commensal flora enhances host defense against bacterial pneumonia by adding interleukin 17 A(IL17A) and regulating GM-CSF signaling (Fig. 5) [74]. One study reported that excessive airway response syndrome in germ free mice was closely associated with T helper 2 (Th2) cells, cytokine (IL-4 and IL-5), and IgE levels in the lungs [75]. It has been shown that in antibiotic-treated mice, respiratory viral infections result in a substantial increase in mortality, which is correlated with reduced abundance of Treg cells in the respiratory and gastrointestinal tracts [76, 77] .

The study of gut microbiota provides a new reference for exploring the causes of malignant tumors. The escape of intestinal microorganisms and their toxic metabolites, such as surviving gastrointestinal bacteria, cell wall fragments, or protein fragments from bacterial death, occur together and enter the pulmonary circulation through the blood and lymphatic systems, causing an imbalance in the physiological state and influencing specific lung inflammation and tumorigenesis through various active molecules [78]. The bi-directional pulmonary-intestinal axis connects the microbiota of the lung and the intestine, and since they both belong to the mucosal immune system organ (MIS) and are covered by similar mucous membranes, they have similar functions of interaction between the immune system and the microbiota. In addition, they are indirectly linked to the lymphatic and circulatory systems and can therefore affect the immune system locally or cause systemic effects. The intensity of their immune response depends on the location of the first interaction. It has been shown that the clinical response and prognosis of lung and intestinal flora to immunotherapy are closely related to the induction of inflammatory processes and immune checkpoint inhibition [79]. Gut microbiota can affect the occurrence, development and prognosis of lung cancer by acting on the respiratory system through the gut-lung axis. Lymph and blood circulation provide physiological pathways for this process [80]. Protein fragments and cell wall components of dead or surviving bacteria will enter the chyle pool through the intestinal lymphatic system, and then enter the lungs through the blood circulation to activate adaptive immunity, leading to the differentiation and activation of T cells, as well as the activation of macrophages and dendritic cells, thus affecting the occurrence and treatment of lung cancer [81, 82]. Patients with lung cancer treated with antibiotics prior to chemotherapy or immunotherapy have less efficacy and shorter survival compared to subjects who did not receive antibiotics [83]. The intestinal microenvironment has an influential impact on the occurrence, progression and treatment of lung cancer, and intestinal flora can affect the development and metastasis of lung cancer through, regulation of the microecological system, the immune system and metabolites, and intestinal microenvironmental homeostasis plays a key role in the efficacy and prognosis of lung cancer radiotherapy, targeted therapy and immunotherapy.

## Regulation of lung and intestinal micro-immune ecology inhibiting the progression of lung cancer

In the Northern Song Dynasty, it is said in the *Classified Materia Medica from Historical Classics for Emergency* that Tangerine peel decoction is used to treat pulmonary carbuncles caused by Xiben (lung cancer), and is taken with a decoction of gecko, donkey-hide gelatin, deerhorn glue, rhinoceros horn and antelope horn. In the Northern Song Dynasty, a formula book named *Taiping Shenghui Fang* recorded that Sangbaipi decoction was used to treat cough caused by lung cancer, coughing up blood and thick phlegm. In the Ming Dynasty, the literature *Qixiao Liangfang* (miracle cure) recorded that Xiben was treated with Xibendecoction, a formula containing pinellia ternata, cinnamon, ginseng, tetradium ruticarpum, mulberry root bark, lepidium, and prepared liquorice root, which was decocted in water and taken before meals. In the Ming Dynasty, it was recorded in the *Chishui Xuanzhu Quanji* that the Xiben was under the right side of the hypochondrium and developed into pulmonary carbuncle, and it is treated with Jujube paste pill (composed with tangerine peel, balloon flower root, lepidium and jujube). In the Qing Dynasty, *A Brief Guide to Medical Formulas* has written that Xiben is treated with a decoction of helianthus bark and lily with wine. A famous book *The Divine Husbandman’s Herbal Foundation Canon* of the Han Dynasty said that Xiben cannot be treated with moxibustion but must be treated with functional training in adjunct to medicine. Besides, the book *Integration of Acupuncture and Moxibustion* in Qing dynasty records that the Chize acupoint is used to treat shoulder and back pain, numbness, or pain in the limbs. In the Ming Dynasty, the medical book *Lei Jing Tu Yi* says that Xiben is under the right side of the ribs, and is treated with the Chize, Zhangmen, and Zusanli acupoints. *The Source of Acupuncture and Moxibustion* said that Qimen, Zhangmen, and Shouze acupoints can be used to treat lung cancer. In summary, ancient Chinese literature indicates that the symptoms of lung cancer are consistent with the clinical manifestations of lung cancer today, with local manifestations including cough, blood in sputum or coughing up blood, chest pain, and dyspnea, and systemic manifestations of emaciation, anemia, and cachexia [84–87]. In ancient times, the treatment plan for lung cancer was based on Chinese herbal formulas, and supplemented with functional training and acupuncture treatment. Based on the treatment of local symptoms of the lung, emphasis was placed on overall condition, especially the use of medicines that regulate the *Qi* and functions of the gastrointestinal tract, such as ginger, jujube, roasted licorice, hedgehog, and tangerine peel, etc. Moreover, immunity-boosting agents, such as ginseng, ass-hide gelatin, and deerhorn glue, were incorporated.

A growing set of studies suggests that dysregulation of the gut microbiota plays an important role in cancer progression and prognosis, and that changes in gut microbiome have the potential to increase immune checkpoint inhibitor (ICIs)-related adverse effects and severe immune-related toxicities (IrAEs) in patients with advanced metastases from NSCLC and hepatocellular liver cancer (HCC).Treatment with ICIs prior to antibiotics was associated with poor progression free survival (PFS) and overall survival (OS) [40, 88]. Routy et al. demonstrated that transplantation of gut microbiota from lung cancer patients that responded to immunosuppressive therapy (PD-1/PD-L1) into antibiotic-treated mice restored immune response, with bacteria Akkermansia muciniphila and Aliistipes uninctus being highly influential [40]. In addition, dietary supplement and adequate physical exercise are non-pharmacological healthy ways, and different dietary composition may affect gut microbiota structure [64, 89, 90]. Studies have confirmed the connection between high consumption of meat and fat and the development of lung disease and tumors [91–94]. The short-chain fatty acid cycle regulates the immune response through the intestinal flora and contributes to the maintenance of stasis in different organs, including the lungs. Consumption of a high-fiber diet and yogurt, rich in prebiotics and probiotics reduces the risk of lung cancers [95–98]. Numerous clinical studies suggest that the treatment of lung cancer must be considered in the context of the whole organism, with special emphasis on the impact of gastrointestinal micro-ecological regulation on lung cancer treatment.

The theory of “The lung and gastrointestinal tract are external and internal” is an important part of the doctrine of visceral manifestation theory. Qingfeiyin decoction can reduce the viral load, partially recover the colon atrophy caused by H1N1 virus, down-regulate the MAPK, tumor necrosis factor α(TNFα)and other inflammatory pathways, reduce lung injury, and improve the survival rate, which fully reflects the effectiveness of “lung-gut co-treatment”, and this theory has been applied to guide the treatment of refractory lung and intestinal diseases such as COVID-19 and ulcerative colitis, and reliable results have been obtained [99, 100].In the treatment of lung cancer, it should not only consider the lung but also the physiological function of the large intestine, and the treatment of intestinal diseases should take into account the physiological function of the lung [101–104], which is called “lung-gut co-treatment”. The physio-pathological relationship between the lung and gastrointestinal tract was shown in *the Inner Canon of Yellow Emperor* more than 3000 years ago, and modern studies have shown that decoctions named “Xuanbai Chengqi”, “Gegen Qinlian”, and tonic herbs such as ginseng, gardenia, angelica, and astragalus can improve acute lung tissue injury and pathological colon tissue damage caused by lipopolysaccharide (LPS) by regulating lung-intestinal mucosal immune function [105–107].Research on the mechanism of traditional Chinese medicine in the treatment of lung cancer has also made progress. Yiqi Yangyin Decoction (YYD) inhibits the proliferation of NSCLC cells. With the increase of YYD concentration, the expression of cell cycle-related proteins p53 and p21 increased, and the expression of cyclin D1 decreased. YYD inhibited the proliferation of NSCLC cells by inhibiting the EGFR-PI3K-AKT signaling pathway [108]. Feiyiliu Mixture could reduce the phosphorylation of EGFR and down-regulate the expression of cyclin, and up-regulate the level of cleaved Caspase-3 protein to promote cell apoptosis [109]. Therefore, lung cancer treatment should consider modulating intestinal function as well. The concept of lung-gut co-treatment could serve as the foundation for exploration of novel drugs.

## Conclusions

**Fig. 6:**
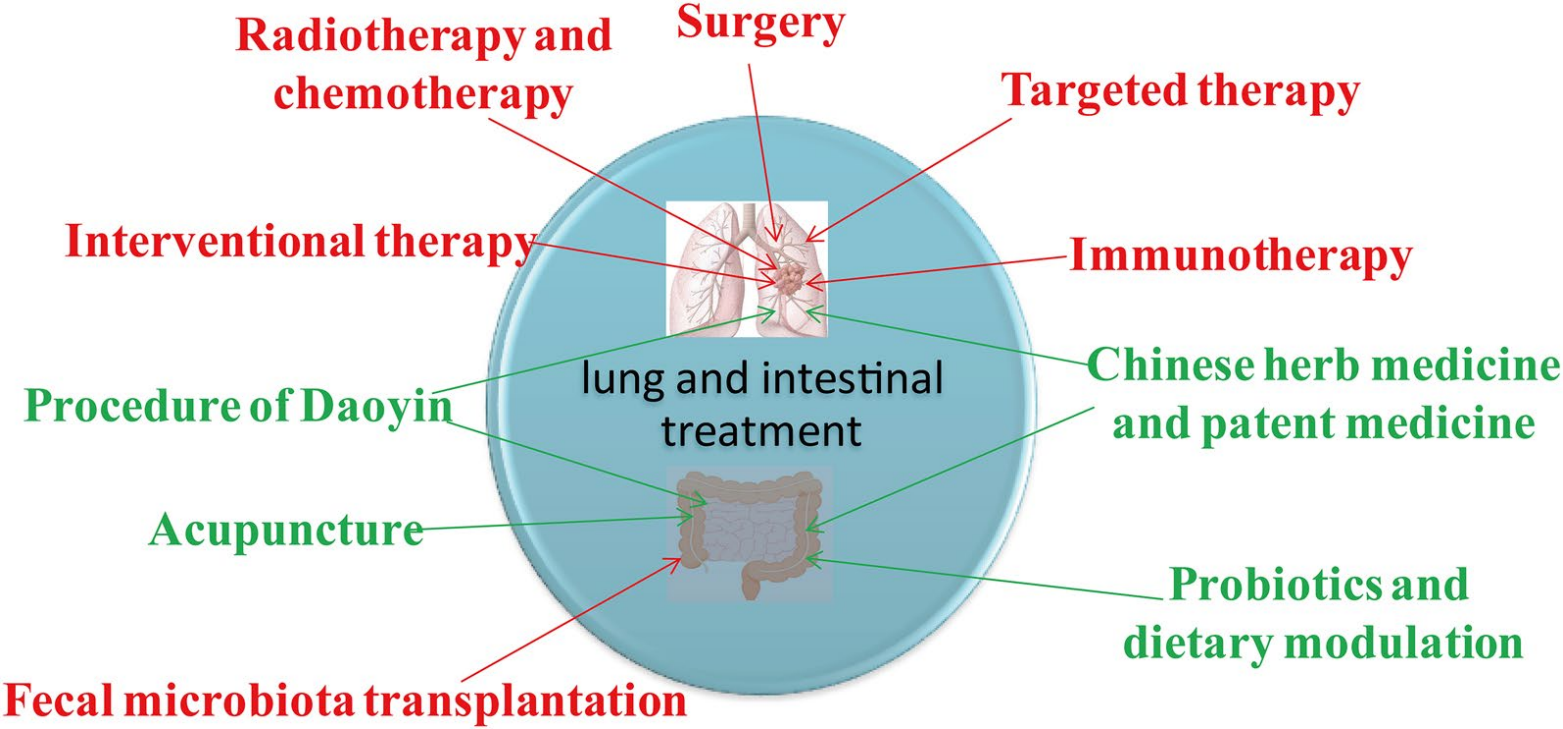
The treatment on lung and intestine is based on the combination of Traditional Chinese and Western medicine. The green font represents TCM therapy, and the red font represents western medicine therapy. The selection of treatment is based on the patient condition

Traditional medicine has elaborated the pathophysiological characteristics of the lung and the gastrointestinal tract, and proposed the theory that the lung and gastrointestinal tract are external and internal. Growing evidence of modern medical research indicates an important and complex interaction between the lung and the intestine, as well as between the intestinal microbiota and host immunity, which is the lung-gut axis. The imbalance of lung and intestinal microecology and immunological barrier is closely related to lung cancer formation, metastasis, and prognosis. Based on the ancient theory of Chinese medicine and the modern findings of the lung-gut axis, we propose that treatment of lung cancer must be “lung-gut co-treatment”. The choice of “lung-gut co-treatment” should be made according to the characteristics of the patient’s disease, symptoms and signs, pathological types and clinical stages. According to the guidelines, surgical treatment is preferred for early stage lung cancer, and postoperative western medical treatment such as radiotherapy and chemotherapy is not needed. Generally, Chinese herb and patent medicine, acupuncture, and Daoyin can be recommended. For patients with advanced stage lung cancer who need radiotherapy and chemotherapy after surgery or are inoperable, interventional therapy, radiotherapy and chemotherapy, targeted therapy and immunotherapy can be selected according to their conditions. Probiotics and dietary modulation can be added. For patients who can not tolerate surgery, radiotherapy, chemotherapy or other western medicine treatment (or have poor efficacy), traditional Chinese medicine treatment should be given priority and combine with fecal microbiota transplantation, probiotics and dietary modulation (Fig. 6). Using “lung-gut co-treatment” to modify intestinal flora and improve the gut-lung immune imbalance provides a new strategy for the comprehensive treatment of lung cancer.

## Data Availability

Not applicable.
